# Systemic Lupus Erythematosus 2014

**DOI:** 10.1155/2014/274323

**Published:** 2014-03-24

**Authors:** Juan-Manuel Anaya, Yehuda Shoenfeld, Ricard Cervera

**Affiliations:** ^1^Center for Autoimmune Diseases Research (CREA), School of Medicine and Health Sciences, Universidad del Rosario, 11001000 Bogota, Colombia; ^2^Méderi Hospital Universitario Mayor, 11001000 Bogota, Colombia; ^3^Zabludowitcz Center for Autoimmune Diseases, Sheba Medical Center, Tel-Hashomer, Sackler Faculty of Medicine, Tel Aviv University, 52621 Tel-Hashomer, Israel; ^4^Department of Autoimmune Diseases, Hospital Clinic, Calle Villarroel 170, Barcelona, Catalonia, Spain

Systemic lupus erythematosus (SLE, lupus) is the prototype of systemic autoimmune disease (AD). Immune system activation in SLE is characterized by exaggerated B-cell and T-cell responses and loss of immune tolerance against self-antigens. Production and defective elimination of antibodies, circulation and tissue deposition of immune complexes, and complement and cytokine activation contribute to clinical manifestations that range from fatigue and joint pain to severe, life-threatening organ damage [1].

In this special issue, nine papers were selected covering important topics of the disease from B lymphocytes (e.g., autoantibodies and B-cell depletion therapy), immunosenescence, and genetics to disease complications such as cardiovascular disease (CVD), preeclampsia, fatigue, and depression.

B lymphocytes are the effectors of humoral immunity, providing defense from pathogens through different functions including antibody production. In the context of ADs, B lymphocytes play an essential role by not only producing autoantibodies but also functioning as antigen presenting cells and as a source of cytokines as pointed out by G. J. Tobón and colleagues, who reviewed the functions of B lymphocytes in autoimmunity and ADs with a special focus on their abnormalities in SLE.

We recognize today that disease manifestations are determined by the diversity of autoantibodies appearing in SLE [2, 3]. This explains the different clinical presentations within individuals with SLE. In this issue, E. Cozzani and colleagues reviewed the most important autoantibodies in SLE and their correlation between immunopathological features and clinical aspects. Recommendations for determining anti-nuclear antibodies, anti-double stranded DNA antibodies, specific antibodies, and validation of methods have been published elsewhere [4].

Given that autoantibody production is the hallmark of SLE, it is not surprising that B-cell depletion therapy is a promising therapeutic option in the management of SLE. Rituximab (RTX), a chimeric anti-CD20 monoclonal antibody, has been used off-license in the management of severe refractory SLE since 2002. In this special issue, F. Bonilla-Abadía and colleagues report the results of a retrospective and descriptive observational study of patients with SLE refractory to conventional treatment who were treated with RTX as remission induction therapy and maintenance. They observed a significant reduction in the conventional immunosuppressive drug dose and the number of relapses of disease suggesting that RTX could be effective and safe in patients with SLE refractory to conventional therapy.

An important matter about SLE for 2014 will be the progress and even release of results of the ongoing trials with the new biological therapies including epratuzumab, a humanized anti-CD22 monoclonal antibody, and subcutaneous belimumab, a human monoclonal antibody that inhibits B-lymphocyte stimulator, as well as the investigator-initiated trials with RTX (i.e., RITUXILUP and Ring) [5].

Senescence is a normal biological process that occurs in all organisms and involves a decline in cell functions. In the context of the immune system, this phenomenon is known as immunosenescence and refers to the immune function deregulation. A complete review about this topic is also presented in this issue.

ADs are observed in genetically susceptible individuals in whom their clinical expression is modified by permissive and protective environments occurring over time [6]. A plethora of new susceptibility genetic variants for ADs has emerged. As per the case of SLE, more than 100 loci have been replicated by several independent studies that modify the risk to acquire the disease. In this issue, J. E. Molineros and colleagues from Oklahoma Medical Research Foundation report a replication study in which 22 recently identified SLE susceptibility genes were strongly associated with Malaysians.

In order to better understand the genetic basis of SLE that might be due to natural selection, P. S. Ramos and colleagues report an original study showing positive selection at several SLE-associated loci. Their results “provide corroborating evidence in support of recent positive selection as one mechanism underlying the elevated population frequency of SLE risk loci and should stimulate future research that integrates signals of natural selection to help identify functional SLE risk alleles.”

Concerning environmental factors involved in SLE induction, we are surprised each time by novel and modern factors. One of the last ones belongs to the ASIA (Autoimmune Syndrome Induced by Adjuvants). In this sense, human papillomavirus quadrivalent (types 6, 11, 16, and 18) vaccine, recombinant (Gardasil), which became almost mandatory in many countries, has aluminum as adjuvant. It is not surprising that 6 cases of SLE following Gardasil vaccination were reported recently [7]. Further studies on this topic are expected to come to light in 2014.

CVD is a major concern in patients with SLE, whose disease expression varies depending on several factors including ancestry. J. Amaya-Amaya and colleagues report a high rate of CVD in Latin American patients with SLE and encourage preventive population strategies aimed to facilitate the suppression of cigarette smoking and coffee consumption to the tight control of dyslipidemia and other modifiable risk factors for such complication.

One of the obstetric complications of SLE is preeclampsia. A. Schramm and M. E. B. Clowse, after providing an overview of the pathogenesis of preeclampsia, preeclampsia in lupus pregnancies, and previous trials for prevention of preeclampsia with aspirin treatment, recommend low-dose aspirin administration for all lupus patients starting prior to 16 weeks of gestation. Further clinical trials are needed to confirm this recommendation.

Patients with SLE report higher levels of cognitive difficulties, depression, pain, and fatigue. R. Fonseca and colleagues report a case-control study in which significant lower scores in quality-of-life dimensions related to physical impairment were found in patients with SLE as compared with controls. Authors suggest that unexplained fatigue in SLE may signify an early sign of immune activation flare-up.

We hope readers of Autoimmune Diseases will enjoy this special issue and be encouraged to translate this new knowledge into practice.

*Juan-Manuel Anaya* *Yehuda Shoenfeld* *Ricard Cervera*
